# Intrauterine Growth Restriction and Patent Ductus Arteriosus in Very and Extremely Preterm Infants: A Systematic Review and Meta-Analysis

**DOI:** 10.3389/fendo.2019.00058

**Published:** 2019-02-08

**Authors:** Eduardo Villamor-Martinez, Mohammed A. Kilani, Pieter L. Degraeuwe, Ronald I. Clyman, Eduardo Villamor

**Affiliations:** ^1^Department of Pediatrics, School for Oncology and Developmental Biology (GROW), Maastricht University Medical Center (MUMC+), Maastricht, Netherlands; ^2^Cardiovascular Research Institute and Department of Pediatrics, University of California, San Francisco, San Francisco, CA, United States

**Keywords:** small for gestational age, growth restriction, patent ductus arteriosus, very preterm infant, meta-analysis, meta-regression

## Abstract

It is generally accepted that intrauterine growth restriction (IUGR) increases morbidity and mortality among very preterm neonates. However, evidence is hampered by the widespread practice of using the terms small for gestational age (SGA) and IUGR as synonyms. We conducted a systematic review of studies reporting on the association between IUGR/SGA and patent ductus arteriosus (PDA). PubMed/MEDLINE and EMBASE databases were searched. Of 993 studies reviewed, 47 (50,790 infants) were included. Studies were combined using a random effects model and sources of heterogeneity were determined by subgroup and meta-regression analyses. Meta-analysis of all included studies showed a significantly reduced risk of PDA in the SGA/IUGR group with an odds ratio (OR) of 0.82, and a 95% confidence interval (CI) of 0.70 to 0.96 (*p* = 0.015). Of the 47 studies, only 7 used a definition for growth restriction that went beyond birth weight (BW) for gestational age (GA). When pooled, meta-analysis could not demonstrate a significant effect size (OR 1.31, 95% CI 0.75 to 2.27, *p* = 0.343). Moreover, the significantly reduced risk of PDA was found in the 25 studies defining SGA as BW <10th percentile (OR 0.81, 95% CI 0.66 to 0.98, *p* = 0.032), but not in the 6 studies defining SGA as BW <3rd (OR 1.09, 95% CI 0.70 to 1.71, *p* = 0.694), or in the 27 studies using a more refined definition of PDA (i.e., hemodynamically significant PDA or PDA requiring treatment, OR 0.87, 95% CI 0.72 to 1.04, *p* = 0.133). In addition, we found that GA was significantly higher in the SGA/IUGR group (18 studies, mean difference 0.63 weeks, 95% CI 0.24 to 1.03, *p* = 0.002). Meta-regression analysis confirmed the correlation between this difference in GA and PDA risk. In summary, we observed marked heterogeneity across studies in the definition of growth restriction and PDA, and we found differences between the control and growth-restricted groups in relevant baseline characteristics, such as GA. Therefore, our meta-analysis could not provide conclusive evidence on the association between growth restriction and PDA.

## Introduction

The ductus arteriosus (DA) of very preterm infants is less likely to close spontaneously as part of the transition to extrauterine life and, consequently, the incidence of patent DA (PDA) is inversely related to gestational age (GA) at birth (1–4). Intrauterine or fetal growth restriction (IUGR/FGR) is commonly recognized as an additional major risk factor for mortality and morbidity in very preterm infants (5–11). One of the conditions that IUGR has been associated with is PDA (12–14), but the evidence supporting this association is scarce and has not been systematically reviewed. Some studies even suggest that IUGR may protect against PDA (15–18).

A common problem of studies assessing the potential association of growth restriction with adverse neonatal outcomes is that they do not differentiate between small for gestational age (SGA) and IUGR, even though the two terms are not synonymous (7–10). SGA is a statistical definition based on birth weight (BW), with the 10th percentile as the most commonly used threshold. The term SGA differs from IUGR principally because it also encompasses constitutionally small but healthy infants at lower risk of abnormal perinatal outcome. On the other hand, growth restricted infants who have a BW above the 10th percentile may be falsely classified as normally grown (6–10).

The most common cause of IUGR is placental insufficiency leading to fetal hypoxia and undernutrition (19). Whether the normal development of the DA is affected by these pathological conditions remains largely unknown. Experimental animal studies showed that reactivity of DA is impaired by exposure to chronic fetal hypoxia (20, 21). King et al. showed histological evidence of accelerated DA maturation in very preterm infants exposed to chronic intrauterine stress, leading to the hypothesis that this may have resulted in earlier postnatal DA closure (22). In contrast, Ibara et al. described alterations in the DA of preterm infants with IUGR (23). These include fragmentation, coagulation and necrosis of the internal elastic lamina, as well as hemorrhage with necrosis and loosening of elastic fibers and muscles in the tunica media (23). These ductal changes may explain why hemodynamically significant PDA (hsPDA) has been reported to occur more frequently and at an earlier postnatal age in very preterm infants with IUGR (12, 13).

We aimed to carry out a systematic review of observational studies reporting on the association between IUGR/SGA and PDA. We paid particular attention to how the criteria used to define growth restriction and PDA affected the potential association between the two conditions. We also analyzed the role of potential confounders, such as GA and rate of respiratory distress syndrome (RDS).

## Methods

The methodology of this study is based on that of earlier studies of our group on chorioamnionitis and various morbidities (24–26) and PDA and platelet counts (27). The study is reported according to the Preferred Reporting Items for Systematic Reviews and Meta-Analyses (PRISMA) guidelines (28). The PRISMA checklist is included in the Supplementary Material. We developed a protocol a priori defining the objectives, methods, inclusion criteria and approach to assessing study quality.

### Data Sources and Search Strategy

We performed a comprehensive systematic literature search using the PubMed/MEDLINE and EMBASE databases. The first search was performed on October 1st, 2016. Automated alerts were used during the elaboration of the review to maintain the search up to date. The search strategy was as follows for PubMED: (PDA OR ductus arteriosus) AND (preterm OR premature) AND (IUGR OR growth restriction OR growth retardation OR restricted growth OR fetal growth OR fetus growth OR reduced growth OR prenatal growth OR placental dysfunction OR placental insufficiency OR chronic hypoxia OR chronic hypoxemia OR small for gestational age OR small for date OR SGA). We used a similar strategy for EMBASE. There were no language preferences set. Reference lists of relevant primary and review articles were searched for additional studies. The “cited by” function in Web of Science and Google Scholar was also used to expand the search parameters and ensure that all relevant studies were found.

### Study Selection

We included studies which reported on a growth restricted (SGA, IUGR, FGR) group and a comparison group, studied (very and extremely) preterm infants, and reported primary data that could be used to measure the association of SGA/IUGR and PDA. To assess relevance, two reviewers (MAK, PLD) screened the results of the searches and applied inclusion criteria using a structured form. Disagreements were resolved through discussion or in consultation with a third reviewer (EV).

### Data Extraction

A predetermined designed data extraction form was used. Data was extracted from relevant studies by two investigators (MAK, PLD). Accuracy and completeness of the data extraction was then assessed by two other investigators (EVM, EV). Data extracted from each study included citation information, country where research was conducted, language of publication, study design, objectives, inclusion/exclusion criteria, definitions of SGA/IUGR and PDA, patient characteristics [including GA and birth weight (BW)], and results (including raw numbers and adjusted analyses on SGA/IUGR and PDA where available). When studies assessed PDA at several time points, we used the PDA incidence at the last time of assessment for data analysis.

### Quality Assessment

We assessed study quality using the Newcastle-Ottawa Scale for assessing the quality of non-randomized studies in meta-analyses. This scale allocates points for quality in the domains of selection (0–4 points), comparability (0–2 points), and outcome/exposure (0–3 points), for a total of 0–9 points. The process was carried out by two reviewers independently (EVM and EV). Discrepancies were resolved through discussion.

### Statistical Analysis

Studies were pooled and analyzed using comprehensive meta-analysis V 3.0 software (CMA, RRID:SCR_012779, Biostat Inc., Englewood, NJ, USA). For dichotomous outcomes, the odds ratio (OR) with 95% confidence interval (CI) was calculated from the data provided in the studies. For continuous outcomes, the mean difference (MD) with 95% CI was calculated. When studies reported continuous variables as median and range or interquartile range, the mean and standard deviation were estimated using the method of Wan et al. (29).

Summary statistics were calculated with a random-effects model because of anticipated heterogeneity. This model accounts for variability between and within studies. Subgroup analyses were used to analyze sources of heterogeneity. They were based on the mixed-effects model (30). In this model, a random effect model is used to pool studies within each subgroup and a fixed effect model is used to combine the subgroups and generate the summary effect. We assumed a common among-study variance component (tau-squared) across subgroups.

We determined a priori that we would create subgroups for SGA/IUGR definition, PDA definition, and for studies which only included extremely preterm infants (GA < 28 weeks). We also decided to carry out meta-analyses of the following covariates in the SGA and control groups: GA, BW, rate of RDS, and rate of antenatal corticosteroids (ACS). Statistical heterogeneity was assessed using Cochran's Q statistic and I^2^ statistic, which is a derivative from Q and demonstrates the chance of total variation resulting from heterogeneity beyond chance (30). A univariate random-effects meta-regression (method of moments) was used to investigate the role of GA, rate of RDS, and rate of ACS in explaining differences in effect sizes among studies (30). Meta-regression was also used to compare subgroups. Publication bias was evaluated by using Egger's regression test and through visual inspection of funnel plots. We considered a probability value of less than 0.05 (0.10 for heterogeneity) as statistically significant.

## Results

### Included Studies

We identified 993 potentially relevant studies from which 47 (50,790 patients, 7,860 SGA/IUGR cases, 17,300 PDA cases) met the inclusion criteria (5, 12, 13, 15–18, 31–70). The PRISMA search diagram is depicted in Supplementary Figure 1 and the main characteristics of the included studies are shown in Supplementary Table 1.

While all studies provided data to measure the association between SGA/IUGR and PDA, only 3 of the studies were primarily designed to assess this association (13, 32, 69). Twenty-three studies reported on risk factors for PDA, and IUGR/SGA was one of the risk factors considered (Supplementary Table 1). Twenty-one studies examined the outcomes of IUGR/SGA infants, and PDA was one of the outcomes studied (Supplementary Table 1).

**Figure 1:**
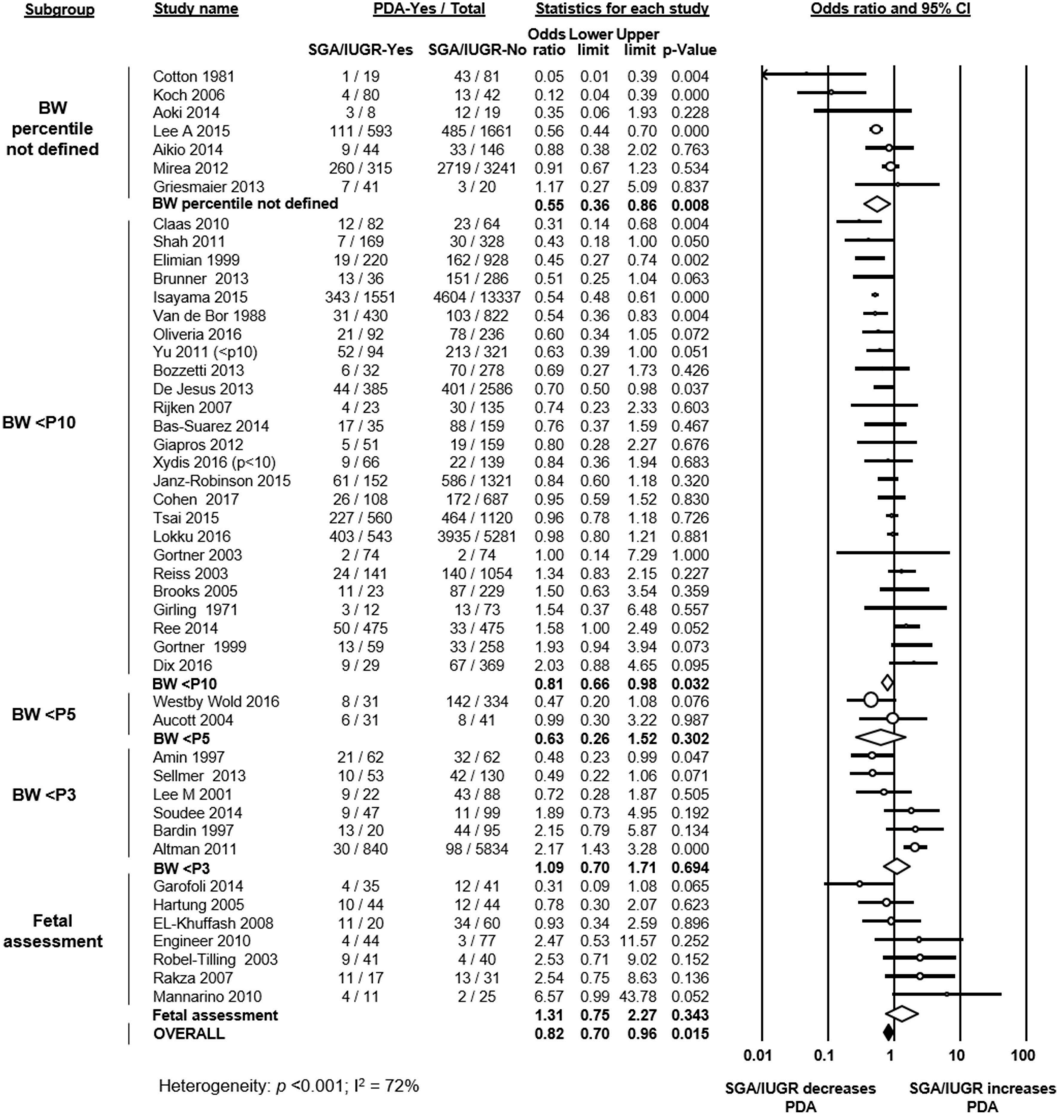
Meta-analysis of the association of small for gestational age (SGA)/intrauterine growth restriction (IUGR) and patent ductus arteriosus (PDA). CI: confidence interval; BW: birth weight; <P10: BW lower than 10th percentile; <P5: BW lower than 5th percentile; <P3: BW lower than 3rd percentile.

Forty studies defined growth restriction based on BW below a determined percentile for GA (Figure 1). From these studies, 25 used the 10th percentile, 2 studies used the 5th percentile, and 6 studies used the 3rd percentile (or −2 standard deviations) (Figure 1). Seven studies did not specify which criteria or percentile was used to define growth restriction (Figure 1). One study used an ultrasound estimated fetal weight chart (48). Six studies used customized BW charts, which adjusted for factors such as sex, ethnicity, socioeconomic status, or parity (15, 17, 35, 50, 57, 63). Thirty-three studies used population-based BW charts (Supplementary Table 1). Eighteen of these 33 studies used sex-specific charts, and 15 used charts that did not differentiate by sex.

Seven studies used a definition of growth restriction that went beyond the use of BW for GA and that included fetal assessment. Of these, 2 studies used a definition of deviation of fetal growth (13, 61), and in 5 studies fetal growth restriction was defined through the presence of an abnormal Doppler result (absent end-diastolic flow in the umbilical artery) (12, 41, 44, 46, 47).

**Figure 2:**
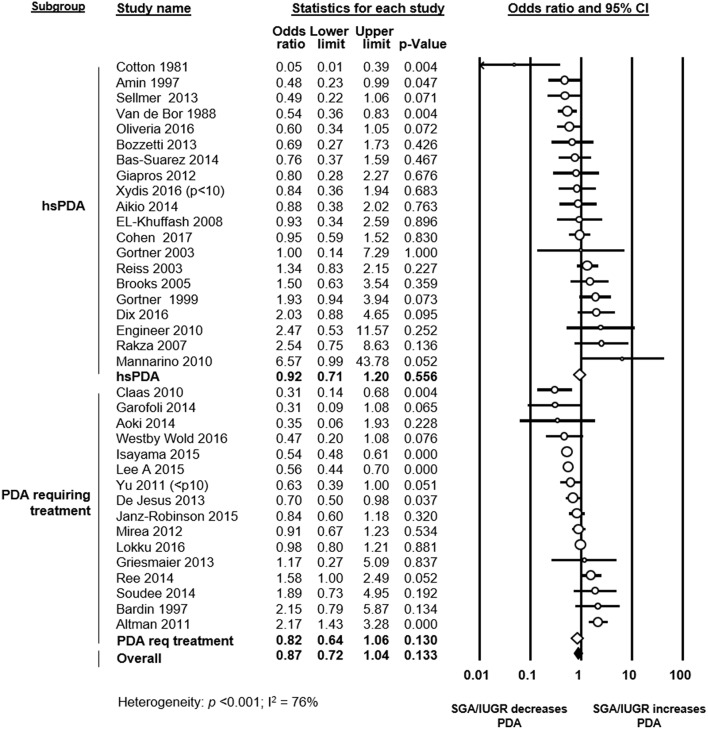
Subgroup meta-analysis of studies using a definition of significant patent ductus arteriosus (PDA) (i.e., hemodynamically significant PDA or PDA requiring treatment). Analysis on the association of growth restriction and PDA. Req: requiring; CI: confidence interval; SGA: small for gestational age; IUGR: intrauterine growth restriction; hsPDA: hemodynamically significant PDA.

The assessment of PDA was based solely on clinical criteria in one study (31), whereas the other studies used heart ultrasound or ultrasound combined with clinical criteria. Thirteen studies defined PDA as significant or hemodynamically significant PDA, and 14 studies defined PDA based on the necessity of treatment (Figure 2 and Supplementary Table 1). Two studies defined all ductal shunts, including the small ones, as PDA (Supplementary Table 1). In 7 studies a definition or definition criteria for PDA was not specified (Supplementary Table 1).

### Quality Assessment

The quality of each study was assessed using the Newcastle-Ottawa Scale and is summarized in Supplementary Table 2. Twelve studies scored 6 points (out of 9), 24 studies scored 7 points, and 11 studies scored 8 points. Studies were downgraded in quality mostly for not adjusting/matching for confounders (*k* = 44), for not clearly defining SGA/IUGR (*k* = 7), and for not clearly defining PDA (*k* = 9).

### Meta-Analysis Results

As shown in Figure 1, meta-analysis of all 47 included studies found a significant negative association between being SGA/IUGR and developing PDA (OR 0.82, 95% CI 0.70–0.96). Neither visual inspection of the funnel plot (Supplementary Figure 2) nor Egger's regression test (*p* = 0.079) suggested publication bias. The association between growth restriction and PDA remained negative and significant when only evaluating studies which defined SGA through BW for GA (40 studies, OR 0.79, 95% CI 0.67–0.93). We further divided this group of studies according to the cut-off percentile. In the meta-analyses of studies using the 10th percentile (25 studies, OR 0.81, 95% CI 0.66–0.98) and studies where the percentile was not reported (7 studies, OR 0.55, 95% CI 0.36–0.86) the significant negative association between SGA and PDA was maintained (Figure 1). In contrast, meta-analyses of studies using the 5th percentile (2 studies, OR 0.63, 95% CI 0.26–1.52), and meta-analysis of studies using the 3rd percentile (6 studies, OR 1.09, 95% CI 0.70–1.71) could not demonstrate a significant association between being SGA and PDA (Figure 1). When examining the subgroup of studies that used a definition for IUGR that went beyond BW for GA (i.e., presence of abnormal Doppler or assessment of fetal growth), meta-analysis could not find a significant association between IUGR and PDA (7 studies, OR 1.31, 95% CI 0.75–2.27, Figure 1). When further subdividing by definition of IUGR, the association remained non-significant for the 5 studies using the abnormal Doppler criteria (OR 1.67, 95% CI 0.71–3.96) and for the 2 studies assessing fetal growth (OR 0.89, 95% CI 0.24–3.38).

To evaluate the role of PDA definition, we performed a further meta-analysis including only studies which defined PDA as hemodynamically significant or PDA requiring treatment. This meta-analysis could not find a significant association between SGA/IUGR and PDA in either subgroup (hsPDA: OR 0.92, 95% CI 0.71–1.20; PDA requiring treatment: OR 0.82, 95% CI 0.64–1.06), or when combining the two subgroups (OR 0.87, 95% CI 0.72–1.04, Figure 2).

**Figure 3:**
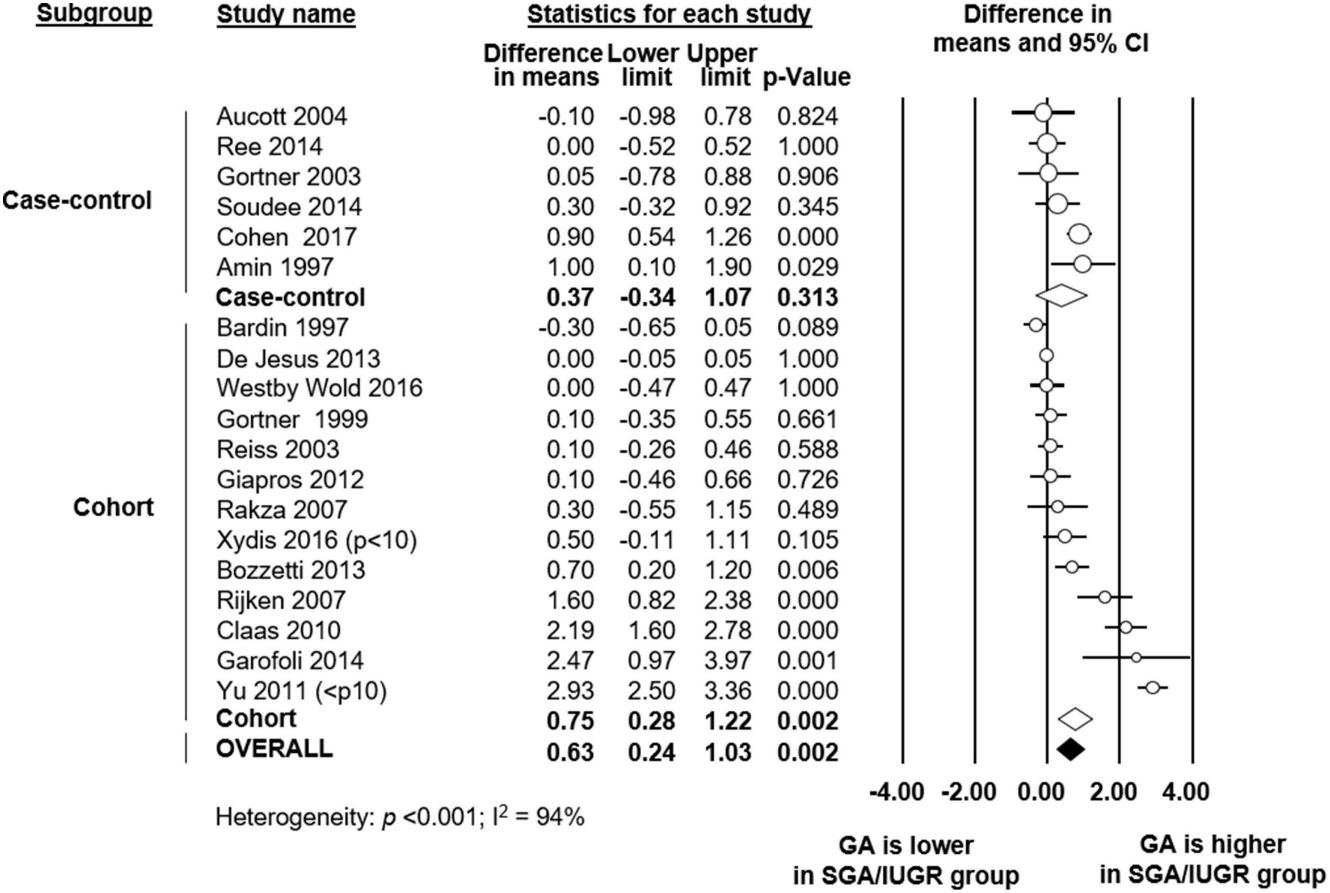
Meta-analysis of mean difference in gestational age (GA) between growth restricted group and control. CI: confidence interval; SGA: small for gestational age; IUGR: intrauterine growth restriction.

To explore sources of heterogeneity, we carried out several additional meta-analyses of covariates, subgroup analyses and meta-regression analyses. Firstly, we examined the role of GA as a confounder in the association between SGA/IUGR and PDA. Six case-control and 13 cohort studies reported data on GA in the SGA/IUGR and the control group. Meta-analysis found that infants in the SGA/IUGR group were born significantly later (18 studies, MD 0.63 weeks, 95% CI 0.24 to 1.03, Figure 3) than infants in the control group. Although this effect was more pronounced in cohort studies, it was also found in some case-control studies (Figure 3). The difference in GA between the SGA and the control group was particularly marked in the 3 studies (16, 33, 49) that only used BW as inclusion criterion. When these three studies were pooled, meta-analysis showed a MD in GA of 2.23 weeks (95% CI 1.66–2.79). Meta-analysis also found, as expected for a condition defined primarily using BW, that infants in the SGA group were significantly lighter at birth (18 studies, MD −379 g, 95% CI −452 to −306, Table 1). Both meta-analyses of GA and BW showed high statistical heterogeneity (Table 1).

**Table 1:** Meta-analyses of confounding variables.

**Meta-analysis**	***k***	**Effect size**	**95% CI**	***p***	**Heterogeneity**	
					***I^**2**^***	***p***
Gestational age (MD)	18	0.63 weeks	0.24 to 1.03	0.002	94%	<0.001
Birth weight (MD)	18	−379 g	−452 to −306	<0.001	97%	<0.001
Antenatal corticosteroids (OR)	17	1.18	0.94 to 1.49	0.159	59%	0.001
Respiratory distress syndrome (OR)	23	0.77	0.60 to 0.98	0.035	78%	<0.001

*CI: confidence interval; k: number of studies; MD: mean difference (growth restricted group minus control group); OR: odds ratio*.

**Figure 4:**
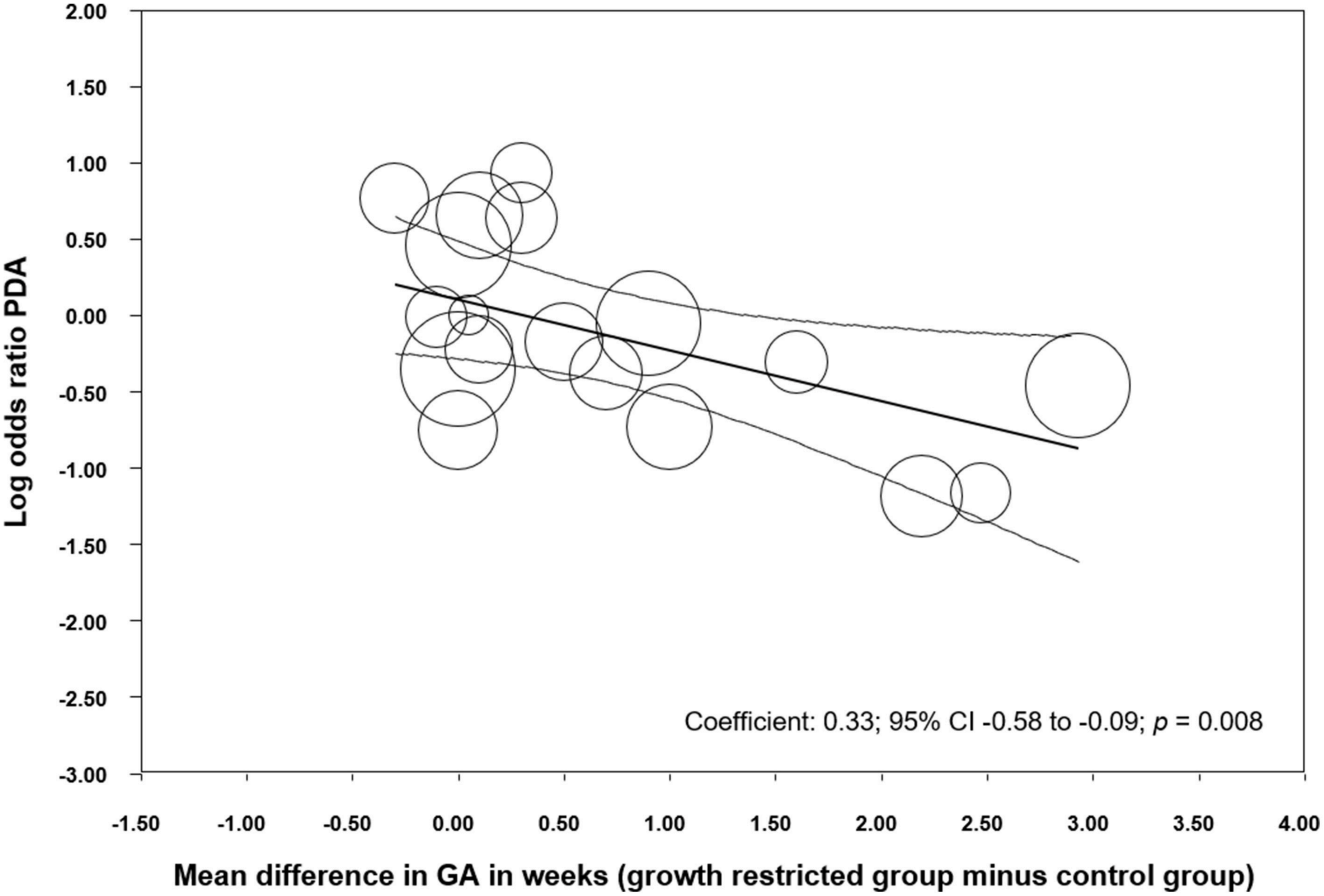
Meta-regression of mean difference in gestational age (GA) between growth restricted and control groups and risk of developing patent ductus arteriosus (PDA). CI: confidence interval.

We performed additional sensitivity analysis to investigate the possible influence of the differences in GA between the SGA/IUGR and the control group on the association between PDA and growth restriction. Meta-analysis of studies where infants with growth restriction were born more than 0.5 weeks later than control infants, found a protective effect of being growth restricted against PDA (Table 2). In contrast, meta-analysis of studies where growth-restricted infants were born < 0.5 weeks later than control infants could not find a significant effect of growth restriction on PDA (Table 2). Meta-regression confirmed that the difference in effect size between these subgroups was statistically significant (*p* = 0.009, Supplementary Figure 3). When we grouped the studies according to the criteria of having or not having a statistically significant difference (*p* < 0.05) in GA between the SGA/IUGR and the control group, we found similarly that growth restriction was only a protective factor for PDA when growth-restricted infants were born significantly later than control infants (Table 2), and this difference between subgroups was also confirmed through meta-regression (*p* = 0.008, Supplementary Figure 4). In addition, meta-regression showed a significant linear correlation between MD in GA and risk of PDA (Figure 4). Sensitivity analysis of studies which only included infants with a GA <28 weeks or a <1,000 g BW found a significant association between SGA/IUGR and PDA (Table 2, Supplementary Figure 5).

**Table 2:** Subgroup analyses.

**Subgroup criteria**	***k***	**OR**	**95% CI**	***p***	**Heterogeneity**	
					***I^**2**^***	***p***
SGA/IUGR-group had a MD in GA < 0.5 weeks compared to control	10	1.19	0.82–1.71	0.357	57%	0.014
SGA/IUGR-group had a MD in GA ≥0.5 weeks compared to control	8	0.60	0.41–0.89	0.010	16%	0.303
SGA/IUGR-group did not differ significantly in GA from control (*p* ≥ 0.05)	11	1.15	0.81–1.63	0.432	52%	0.022
SGA/IUGR-group did differ significantly in GA from control (*p* < 0.05)	7	0.58	0.38–0.87	0.009	24%	0.244
All infants had GA ≤ 28 weeks or BW ≤ 1,000 g	28	0.81	0.67–0.97	0.020	55%	<0.001
All infants were screened for PDA	16	0.72	0.53–0.97	0.031	53%	0.007
Presence of PDA was assessed in selected infants	7	0.68	0.54–0.87	0.002	55%	0.036

*k: number of studies; OR: odds ratio; CI: confidence interval; SGA: small for gestational age; IUGR: intrauterine growth restriction; GA: gestational age; MD: mean difference; BW: birth weight; PDA: patent ductus arteriosus*.

In another subgroup analysis, we tested whether the studies that screened all infants for PDA (see Supplementary Table 1) showed a different effect size for the association between SGA/IUGR and PDA. As shown in Table 2 and Supplementary Figure 6, universal screening for PDA did not significantly affect the OR of the association between SGA/IUGR and PDA (meta-regression *p* = 0.990). Finally, we performed additional meta-analyses to investigate whether the rate of use of ACS and the rate of RDS were different in the SGA/IUGR and the control group. As shown in Table 1, rate of ACS use was not significantly different but RDS rate was significantly reduced in the SGA/IUGR group. However, the significant negative association between SGA/IUGR and RDS was only observed for the subgroup of studies using the 10th percentile definition of SGA (OR 0.70, 95% CI 0.51–0.95). Meta-regression could not demonstrate a significant correlation between the effect size of the association between SGA/IUGR and PDA and the effect size of the association between SGA/IUGR and RDS (*p* = 0.287, R^2^ analog: 0.13, Supplementary Figure 7).

## Discussion

Meta-analysis of observational studies presents challenging methodological issues involving differences in the design of the studies (i.e., cohort and case-control), assessment of exposure and outcomes, and control for potential confounders (24, 71–73). One of the main difficulties in understanding the relationship between IUGR and PDA is the lack of agreement among clinicians and investigators as to what defines these two clinical entities. In the present meta-analysis, we observed a significantly reduced rate of PDA in the SGA/IUGR group when all the studies were pooled. However, analysis of the subgroups with a more refined definition of either growth restriction or PDA could not confirm the association between the two conditions. In addition, we detected that some studies may be biased by including infants with lower GA in the control group.

Very recently, using the Delphi procedure, a consensus definition of neonatal growth restriction was reached by an international panel of pediatric leaders in the field (10). It was proposed to use the term “growth restriction in the newborn” to differentiate neonatal growth restriction from fetal growth restriction and SGA because, despite the overlapping among these terms, infants defined by them are not the same (10). The consensus proposed that the “use of a unique term will promote clarity in the categorization of infants, both in clinical practice and research, and will prevent conflation and confusion with SGA” (10). Growth restriction in the newborn was defined by a BW below the 3rd percentile on population-based or customized growth charts or at least 3 out of 5 of the following: BW <10th percentile; head circumference <10th percentile; length <10th percentile; prenatal diagnosis of FGR; and maternal pregnancy information (e.g., hypertension or preeclampsia) (10). Therefore, a BW below the 10th percentile is not considered sufficient to define neonatal growth restriction (10). Interestingly, meta-analyses of the studies in which SGA was based on BW below the 10th percentile or percentile was not clearly specified were the only analyses showing a significant reduced risk of PDA in the SGA infants. In contrast, we did not observe a significant association between neonatal growth restriction and PDA when the studies using the 3rd percentile criteria and/or prenatal diagnosis of FGR were pooled. Therefore, when the growth restriction definition was refined, the negative association between growth restriction and PDA was not further observed.

Most studies included in the present meta-analysis used population-based BW references to define SGA infants. These references have been developed with large databases and provide BW percentiles by each GA (74–76). However, BW may not represent intrauterine growth trajectory at a given GA because preterm infants are more likely to be growth-restricted. Thus, the 10th or the 3rd percentile of the BW reference in very preterm infants are substantially lower than the corresponding percentiles of the ultrasound-based fetal weight reference (75). Consequently, it has been suggested that, when compared to ultrasound-based fetal weight references, population BW references significantly under-diagnose growth restricted infants in very preterm births (74–76). Alternatively, prescriptive and customized BW references have been proposed to improve the detection of growth restricted infants at higher risk of neonatal mortality and morbidity (10, 76). Prescriptive BW references are derived from infants who were not exposed to antenatal risk factors of FGR (76), whereas customized growth charts are population-based growth charts that have been adjusted for factors predicting BW such as maternal height and weight or ethnic group (10).

Besides the definition of the exposure (i.e., growth restriction), the definition of the outcome (i.e., PDA) is also controversial. Clear evidence is lacking for or against many of the current approaches to a PDA in very preterm infants. These uncertainties have resulted in different definitions of what is considered a “significant PDA” as well as in treatment strategies, which range from aggressive management to a more conservative approach, with some suggesting that the PDA is an innocent bystander to adverse outcomes (2, 3, 77–80). In 27 of the 47 included studies, PDA was defined as hemodynamically significant and/or PDA requiring treatment. When these 27 studies with a more refined PDA definition were pooled, meta-analysis could not show an association between significant PDA and neonatal growth restriction. In addition, we detected considerable heterogeneity concerning the time of and the indication for PDA assessment. Studies including only selected patients at high risk for PDA or with clinical findings suggestive of the condition may have different rates of PDA than studies screening all very or extremely preterm infants. However, subgroup analysis and meta-regression could not demonstrate that the OR for the association between SGA/IUGR and PDA is different between the studies with or without universal PDA screening. Unfortunately, the marked heterogeneity of the time of assessment of PDA did not allow us to perform any meaningful subgroup analysis on timing.

Failure to account for significant differences in baseline characteristics between groups in observational studies can lead to biased estimates. The major risk factor for PDA is low GA (1–4). We detected that in a substantial number of the included studies the SGA/IUGR group had a higher GA than the control group (see Figure 3). The difference in GA was particularly marked in the studies using BW, but not GA, as inclusion criterion. These facts may contribute to explain the “protective” effect of growth restriction on PDA development. Meta-regression analysis confirmed the correlation between the difference in GA between the SGA/IUGR and the control group and PDA risk. Moreover, subgroup analyses limited to studies without substantial difference in GA between the SGA and the control group could not find a significant association between SGA/IUGR and PDA. Zeitlin et al. assessed the prevalence of SGA age among 7,766 very preterm infants (GA below 32 weeks) from 11 European countries and observed that this prevalence was lower when GA was under 28 weeks (81). They speculate that this difference may reflect fewer indicated deliveries for growth restriction among extremely preterm infants (81). Etiology of very preterm birth (i.e., GA <32 weeks) can be divided into two main categories: infection/inflammation and dysfunctional placentation (82). However, the distribution of these two etiologies is not homogeneous. Infants with dysfunctional placentation are frequently less preterm and this etiology is strongly associated with growth restriction (82). In addition, very early mortality, which is a competing outcome for PDA, is also more frequent in the more preterm infants. Altogether this may explain the higher presence of older infants in the SGA/IUGR group. Nevertheless, when we performed a subgroup analysis that only included extremely preterm infants (GA <28 weeks), the results did not substantially differ from the meta-analysis including all preterm infants (Table 2, Supplementary Figure 5).

A common classical assumption among neonatologists is that the intrauterine stress associated with IUGR would accelerate lung maturation leading to a reduced rate of RDS (6). However, the evidence supporting this idea is scarce and, as reviewed by Rosenberg (6), even a number of studies reported a significantly increased risk of RDS in very preterm infants with IUGR. The relationship between PDA and RDS in very preterm infants is complex and bidirectional. In many instances, the presence of a hemodynamically significant PDA is suspected only on the basis of respiratory findings, such as increasing requirements for supplemental oxygen or mechanical ventilation (83). Conversely, changes in pulmonary precapillary tone as consequence of RDS evolution and/or therapy can alter the left-to-right PDA shunt (83). Therefore, we aimed to analyze the possible role of RDS on the association between PDA and neonatal growth restriction. Twenty-three studies included in our review reported on RDS rate and, when pooled, we observed that the protective effect against RDS was only present in the subgroup of studies using the definition of SGA based on BW below the 10th percentile. In addition, meta-regression could not demonstrate a significant correlation between the effect size of the association SGA/PDA and the effect size of the association RDS/PDA.

Besides the issue of the heterogeneity of definitions discussed above, our study has other limitations which deserve consideration. Only 3 studies examined growth restriction and PDA as their primary objective (13, 32, 69). Moreover, many cohort studies did not describe the role of GA or other confounders in infants with and without IUGR/SGA, which makes distinguishing the effect of IUGR from that of confounding factors difficult. Finally, for some definitions of SGA/IUGR only a limited number of studies could be included in subgroup analysis. The strengths of our study also deserve mention, including a comprehensive search, a large number of included studies, inclusion and data extraction by several researchers to reduce bias, and analysis of confounders through subgroup analyses and meta-regression.

## Conclusion

The present systematic review and meta-analysis could not provide conclusive evidence on the association between neonatal growth restriction and PDA risk because of marked heterogeneity in definitions of both the insult and the outcome (i.e., significant PDA) as well as group differences in relevant baseline characteristics, such as GA, across the studies. An improved understanding of factors influencing the natural history of PDA and the risk factors for the condition may promote enhanced precision regarding diagnosis, monitoring, and treatment selection. Further investigation is needed to analyze whether specific conditions leading to neonatal growth retardation such as preeclampsia or pregnancy hypertensive disorders are associated with an altered risk of developing PDA or other complications in very preterm infants.

## Data Availability Statement

The datasets generated and analyzed for this study can be found in the Hardvard Dataverse repository at: https://dataverse.harvard.edu/dataset.xhtml?persistentId=doi:10.7910/DVN/C0HYTD.

## Author Contributions

EV-M collected data and checked data for accuracy, planned and performed the statistical analyses, contributed to the interpretation of the results, drafted the final version of the manuscript, and reviewed and revised the manuscript. MK performed the search, selected studies for inclusion, collected data, contributed to statistical analysis, and drafted an initial version of the manuscript. PD selected studies for inclusion, collected data and supervised data collection, and reviewed and revised the manuscript. RC contributed to interpretation of results and reviewed and revised the manuscript. EV conceptualized and designed the study, contributed to the search, selected the studies for inclusion, supervised data collection, contributed to the statistical analyses and interpretation of the results, and reviewed and revised the manuscript. All authors approved the final manuscript as submitted.

### Conflict of Interest Statement

EV and RC were authors of studies included in the meta-analysis. The remaining authors declare that the research was conducted in the absence of any commercial or financial relationships that could be construed as a potential conflict of interest.
