# Supplementary material for: Intrauterine Growth Restriction and Patent Ductus Arteriosus in Very and Extremely Preterm Infants: A Systematic Review and Meta-Analysis
Source: Front Endocrinol (Lausanne). 2019 Feb 8;10:58. doi: 10.3389/fendo.2019.00058 (PMC6376617; doi:10.3389/fendo.2019.00058)
Supplement: Supplementary file 1 [file Data_Sheet_1.pdf]

Supplementary Material

Intrauterine Growth Restriction and Patent Ductus Arteriosus in Very Preterm Infants: A Systematic Review and Meta-Analysis

Eduardo Villamor-Martinez[1*], Mohammed A. Kilani[1*], Pieter L. Degraeuwe[1], Ronald I. Clyman[2], Eduardo Villamor [1#]

[1] Department of Pediatrics, Maastricht University Medical Center (MUMC+), School for Oncology and Developmental Biology (GROW), Maastricht, The Netherlands

[2] Cardiovascular Research Institute and Department of Pediatrics, University of California San Francisco, San Francisco, CA, USA

*These authors contributed equally to this work.

[#] **Correspondence:**
Eduardo Villamor, MD PhD.
e.villamor@mumc.nl

1 Supplementary Tables

Supplementary Table 1: Characteristics of all included studies.

First author, year	Country	Study type#	Study Perspective*	Prospective/ Retrospective	Infants (n)	Centers (n)	Mean or *median* BW (g)	Mean or *median* GA (weeks)	Definition SGA/IUGR	Growth chart^	Definition of PDA&	PDA screening to all infants	Timing of PDA assessment
Aikio, 2014	Finland	Cohort	PDA	Retro	190	1	1251	28.7	Unclear	Population	hsPDA	Yes	day 2-5
Altman, 2011	Sweden	Cohort	SGA/IUGR	Prosp	6674	NA	2062	33.1	<3rd P	US estimated fetal weight	Treat	NA	NA
Amin, 1997	Canada	Ca-Co	SGA/IUGR	Retro	186	1	936	28.3	<3rd P	Population	hsPDA	NA	NA
Aoki, 2014	Japan	Cohort	PDA	Prosp	27	1	801	26.3	Unclear	Population	Treat	Yes	≤7 days
Aucott, 2004	USA	Ca-Co	SGA/IUGR	Retro	95	1	982	28.4	<5th P	Population	Undef	NA	NA
Bardin, 1997	Canada	Cohort	SGA/IUGR	Prosp	115	1	N	N	<3rd P	Population	Treat	NA	NA
Bas-Suarez, 2014	Spain	Cohort	PDA	Retro	194	1	*1008*	*27.9*	<10th P	Population	hsPDA	Yes	day 2-4
Bozzetti, 2013	Italy	Cohort	SGA/IUGR	Prosp	310	1	*1153*	*29.7*	<10th P	Population	hsPDA	NA	NA
Brooks, 2005	Australia	Cohort	PDA	Prosp	252	1	*893*	*26.0*	<10th P	Population	hsPDA	Yes	NA
Brunner, 2013	Austria	Cohort	PDA	Prosp	322	1	*1174*	*28.5*	<10th P	Population	Any PDA	Yes	day 2-7
Claas, 2010	Netherlands	Cohort	SGA/IUGR	Retro	146	1	*641*	30.	<10th P	Population	Treat	NA	NA
Cohen, 2017	Netherlands	Ca-Co	IUGR/SGA and PDA	Retro	795	1	936	28.7	<10th P	Population	hsPDA	No	≤7 days

First author, year	Country	Study type#	Study Perspective*	Prospective/ Retrospective	Infants (n)	Centers (n)	Mean or *median* BW (g)	Mean or *median* GA (weeks)	Definition SGA/IUGR	Growth chart^	Definition of PDA&	PDA screening to all infants	Timing of PDA assessment
Cotton, 1981	USA	Cohort	IUGR/SGA and PDA	Prosp	100	1	NA	NA	Unclear	Population	hsPDA	NA	NA
De Jesus, 2013	USA	Cohort	SGA/IUGR	Retro	2971	NA	730	*25.0*	<10th P	Population	Treat	NA	NA
Dix, 2016	Netherlands	Cohort	PDA	Prosp	398	1	1312	29.5	<10th P	Population	hsPDA	Yes	NA
Elimian, 1999	USA	Cohort	SGA/IUGR	Prosp	1148	1	NA	NA	<10th P	Customized	Undef	NA	NA
EL-Khuffash, 2008	Ireland	Cohort	PDA	Prosp	80	1	1041	28.3	Doppler		hsPDA	Yes	12-48 h.
Engineer, 2010	UK	Cohort	SGA/IUGR	Retro	121	1	NA	NA	Doppler	Fetal growth	hsPDA	No	NA
Garofoli, 2014	Italy	Cohort	SGA/IUGR	Retro	76	1	1388	31.7	Deviation fetal growth	Fetal growth	Treat	NA	NA
Giapros, 2012	Greece	Cohort	SGA/IUGR	Retro	210	1	1136	28.5	<10th P	Customized	hsPDA	No	NA
Girling, 1971	UK	Ca-Co	PDA	Retro	85	1	NA	NA	<10th P	Population	Sign clinical PDA	Yes	NA
Gortner, 1999	Germany	RCT	SGA/IUGR	Prosp	317	6	1263	29.6	<10th P	Population	hsPDA	NA	NA
Gortner, 2003	Germany	Ca-Co	SGA/IUGR	Prosp	148	1	1748	33.9	<10th P	Population	hsPDA	NA	NA
Griesmaier, 2013	Austria	Cohort	PDA	Prosp	61	1	NA	30.1	Unclear	Population	Treat	Yes	48-72 h

First author, year	Country	Study type#	Study Perspective*	Prospective/ Retrospective	Infants (n)	Centers (n)	Mean or *median* BW (g)	Mean or *median* GA (weeks)	Definition SGA/IUGR	Growth chart^	Definition of PDA&	PDA screening to all infants	Timing of PDA assessment
Hartung, 2005	Germany	Ca-Co	SGA/IUGR	Retro	88	1	1231	NA	Doppler		Undef	NA	NA
Isayama, 2015	Japan, Canada	Cohort	PDA	Retro	14888	NA	1081	28.5	<10th P	Population	Treat	No	NA
Janz-Robinson, 2015	Australia	Cohort	PDA	Retro	1473	10	*944*	*26.5*	<10th P	Population	Treat	No	NA
Koch, 2006	USA	Cohort	PDA	Prosp	122	1	794	26.1	Unclear	Population	Any PDA	Yes	day 3-10
Lee A, 2015	South Korea	Cohort	PDA	Prosp	2254	55	1069	28.3	Unclear	Population	Treat	Yes	NA
Lee M, 2001	USA	Ca-Co	SGA/IUGR	Retro	110	1	1005	27.6	<3rd P	Population	Undef	Yes	NA
Lokku, 2016	Canada	Cohort	PDA	Retro	5824	30	NA	26.9	<10th P	Population	Treat	NA	NA
Mannarino, 2010	Italy	Cohort	PDA	Prosp	36	1	1338	*30.8*	Doppler	Fetal growth	hsPDA	Yes	NA
Mirea, 2012	Canada	Cohort	PDA	Retro	3556	22	NA	26.9	Unclear	Population	Treat	NA	NA
Oliveria, 2016	Portugal	Cohort	PDA	Retro	328	1	*1231*	*30.09*	<10th P	Population	hsPDA	Yes	24-72 h
Rakza, 2007	France	Cohort	IUGR/SGA and PDA	Prosp	48	1	1126	29.1	Deviation fetal growth	Fetal growth	hsPDA	NA	day 1-7
Ree, 2014	Netherlands	Ca-Co	SGA/IUGR	Retro	950	1	1865	33.3	<10th P	Population	Treat	NA	NA
Reiss, 2003	Germany	Cohort	PDA	Prosp	1195	999	1196	28.8	<10th P	Population	hsPDA	NA	NA
Rijken, 2007a	Netherlands	Cohort	SGA/IUGR	Prosp	158	1	1290	29.5	<10th P	Population	Undef	NA	NA

First author, year	Country	Study type#	Study Perspective*	Prospective/ Retrospective	Infants (n)	Centers (n)	Mean or *median* BW (g)	Mean or *median* GA (weeks)	Definition SGA/IUGR	Growth chart^	Definition of PDA&	PDA screening to all infants	Timing of PDA assessment
Robel-Tilling, 2003	Germany	Ca-Co	PDA	Prosp	81	1	1768	32.8	Doppler		Undef	NA	day 1-5
Sellmer, 2013	Denmark	Cohort	PDA	Prosp	183	1	*922*	*27.0*	<3rd P	Population	hsPDA	Yes	day 3
Shah, 2011	USA	Cohort	PDA	Prosp	497	1	983	30.75	<10th P	Customized	Any PDA	Yes	day 3
Soudee, 2014	France	Ca-Co	SGA/IUGR	Retro	146	1	*1068*	*30.0*	<3rd P	Customized	Treat	NA	NA
Tsai, 2015	Taiwan	Cohort	PDA	Retro	1680	21	1052	28.3	<10th P	Population	Undef	NA	NA
Van de Bor, 1988	Netherlands	Cohort	PDA	Prosp	1252	8	1270	30.5	<10th P	Customized	hsPDA	No	NA
Westby Wold, 2016	Norway	Cohort	SGA/IUGR	Retro	365	15	833	26.0	<5th P	Population	Treat	NA	NA
Xydis, 2016	Greece	Cohort	SGA/IUGR	Prosp	205	1	1792	32.9	<10th P	Customized	hsPDA	NA	NA
Yu, 2011	South Korea	Cohort	SGA/IUGR	Retro	415	1	768	26.2	<10th P	Population	Treat	No	NA

SGA: small for gestational age; IUGR: intrauterine growth restriction; PDA: patent ductus arteriosus; BW: birth weight; GA: gestational age; NA: information not available.

#Abbreviations for study design: Ca-Co: case-control study; RCT: randomized control trial.

*Abbreviations for study perspective: PDA: study analyzed SGA/IUGR as a risk factor for PDA; SGA/IUGR: study analyzed PDA as an outcome of SGA/IUGR; IUGR/SGA and PDA: study analyzed the association between SGA/IUGR and PDA as their primary outcome.

^Abbrevaitions for growth chart: Population: population-based growth chart

[&]Abbreviations for definition of PDA: hsPDA: hemodynamically significant PDA; Treat: PDA requiring treatment (pharmacological or surgical); Undef: undefined.

Supplementary Table 2. Details of Newcastle-Ottawa Quality assessment.

Firsty author, year	Selection	Comparability	Outcome /Exposure	Total	Reason for downgrade
Aikio, 2014	3	0	3	6	Unclear definition SGA, No adjustment
Altman, 2011	4	0	3	7	No adjustment
Amin, 1997	4	2	2	8	Unclear definition PDA
Aoki, 2014	3	0	3	6	Unclear definition SGA, No adjustment
Aucott, 2004	4	2	2	8	Unclear definition PDA
Bardin, 1997	4	0	3	7	No adjustment
Bas-Suarez, 2014	4	0	3	7	No adjustment
Bozzetti, 2013	4	1	3	8	No adjustment
Brooks, 2005	4	0	3	7	No adjustment
Brunner, 2013	4	0	3	7	No adjustment
Claas, 2010	4	0	3	7	No adjustment
Cohen, 2017	4	0	3	7	Did not match for GA
Cotton, 1981	3	0	3	6	Unclear definition SGA, No adjustment
De, Jesus, 2013	4	1	3	8	No adjustment
Dix, 2016	4	0	3	7	No adjustment
Elimian, 1999	4	0	2	6	No adjustment, Unclear definition PDA
EL-Khuffash, 2008	4	0	3	7	No adjustment
Engineer, 2010	4	0	3	7	No adjustment
Garofoli, 2014	3	0	3	6	Unclear definition SGA, No adjustment
Giapros, 2012	4	1	3	8	No adjustment
Girling, 1971	4	0	3	7	No adjustment
Gortner, 1999	4	0	3	7	No adjustment
Gortner, 2003	4	1	3	8	Only matched for 1 factor
Griesmaier, 2013	3	0	3	6	No adjustment, Unclear definition SGA
Hartung, 2005	4	1	2	7	Only matched for 1 factor, Unclear definition PDA
Isayama, 2015	4	0	3	7	No adjustment
Janz-Robinson, 2015	4	0	3	7	No adjustment
Koch, 2006	3	0	3	6	Unclear definition SGA, No adjustment
Lee, A, 2015	3	0	3	6	Unclear definition SGA, No adjustment
Lee, M, 2001	4	2	2	8	Unclear definition PDA

Lokku, 2016	4	0	3	7	No adjustment
Mannarino, 2010	3	0	3	6	Unclear definition SGA, No adjustment
Mirea, 2012	3	0	3	6	Unclear definition SGA, No adjustment
Oliveria, 2016	4	0	3	7	No adjustment
Rakza, 2007	4	0	3	7	No adjustment
Ree, 2014	4	1	3	8	Only matched for 1 factor
Reiss, 2003	4	1	3	8	No adjustment
Rijken, 2007	4	0	2	6	No adjustment, Unclear definition PDA
Robel-Tilling, 2003	4	0	2	6	No adjustment, Unclear definition PDA
Sellmer, 2013	4	0	3	7	No adjustment
Shah, 2011	4	0	3	7	No adjustment
Soudee, 2014	4	1	3	8	Only matched for 1 factor
Tsai, 2015	4	1	2	7	Only matched for 1 factor
Van, de, Bor, 1988	4	0	3	7	No adjustment
Westby, Wold, 2016	4	1	3	8	No adjustment
Xydis, 2016	4	0	3	7	No adjustment
Yu, 2011	4	0	3	7	No adjustment

Studies were evaluated as though the association between growth restriction and PDA was the primary outcome.
SGA: small for gestational age; PDA: patent ductus arteriosus.

2 Supplementary Figures

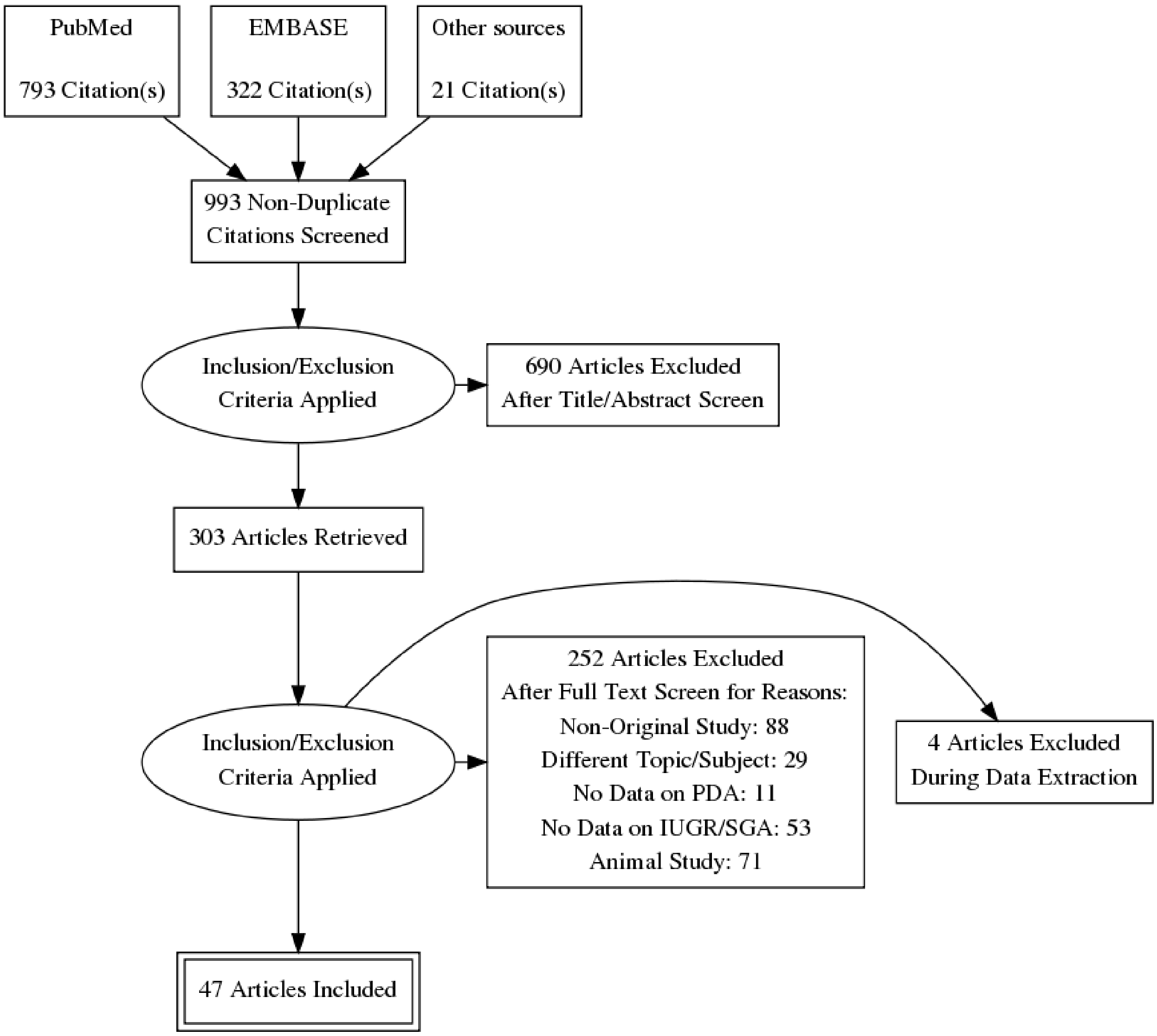

Supplementary Figure 1. PRISMA search diagram of included and excluded studies. PDA: patent ductus arteriosus; SGA: small for gestational age; IUGR: intrauterine growth restriction.

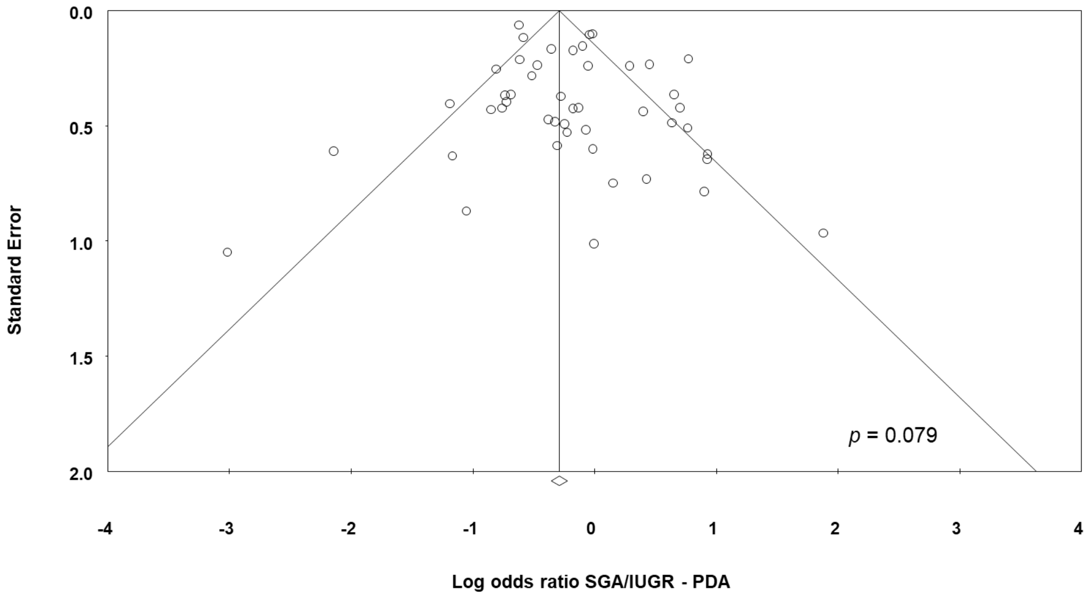

Supplementary Figure 2. Funnel plot for publication bias. SGA: small for gestational age; IUGR: intrauterine growth restriction; PDA: patent ductus arteriosus.

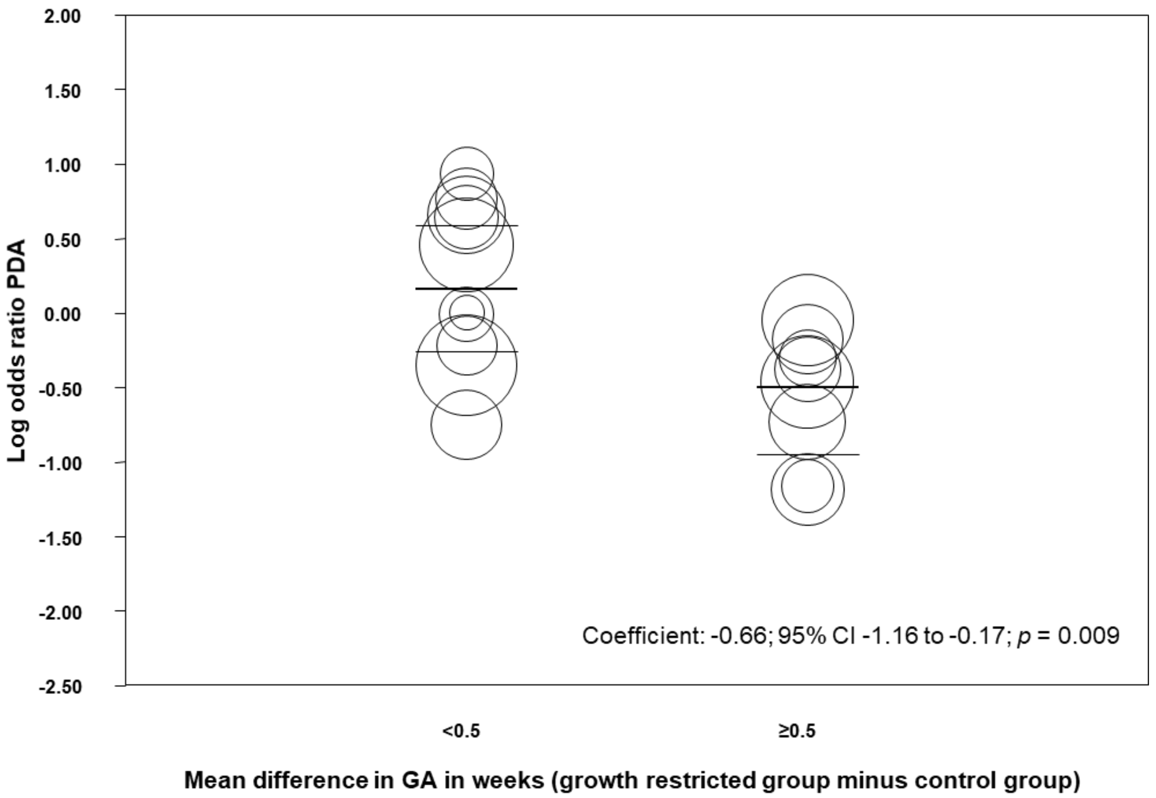

Supplementary Figure 3. Meta-regression comparing studies with a mean difference in gestational age (GA) between groups of < 0.5 weeks vs. studies with a MD in GA of ≥ 0.5 weeks. PDA: patent ductus arteriosus; CI: confidence interval.

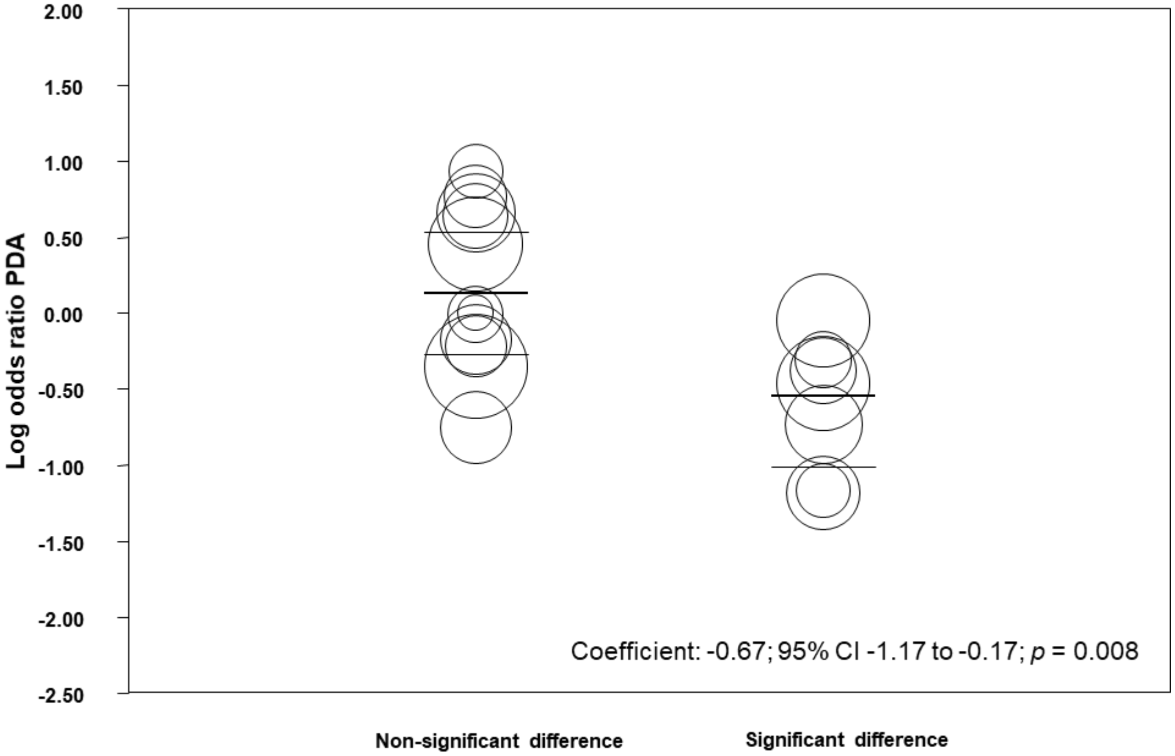

Supplementary Figure 4. Meta-regression comparing studies with a mean difference in gestational age between groups which was non-significant ($p \geq 0.05$) vs. studies where it was significant ($p < 0.05$). PDA: patent ductus arteriosus; CI: confidence interval.

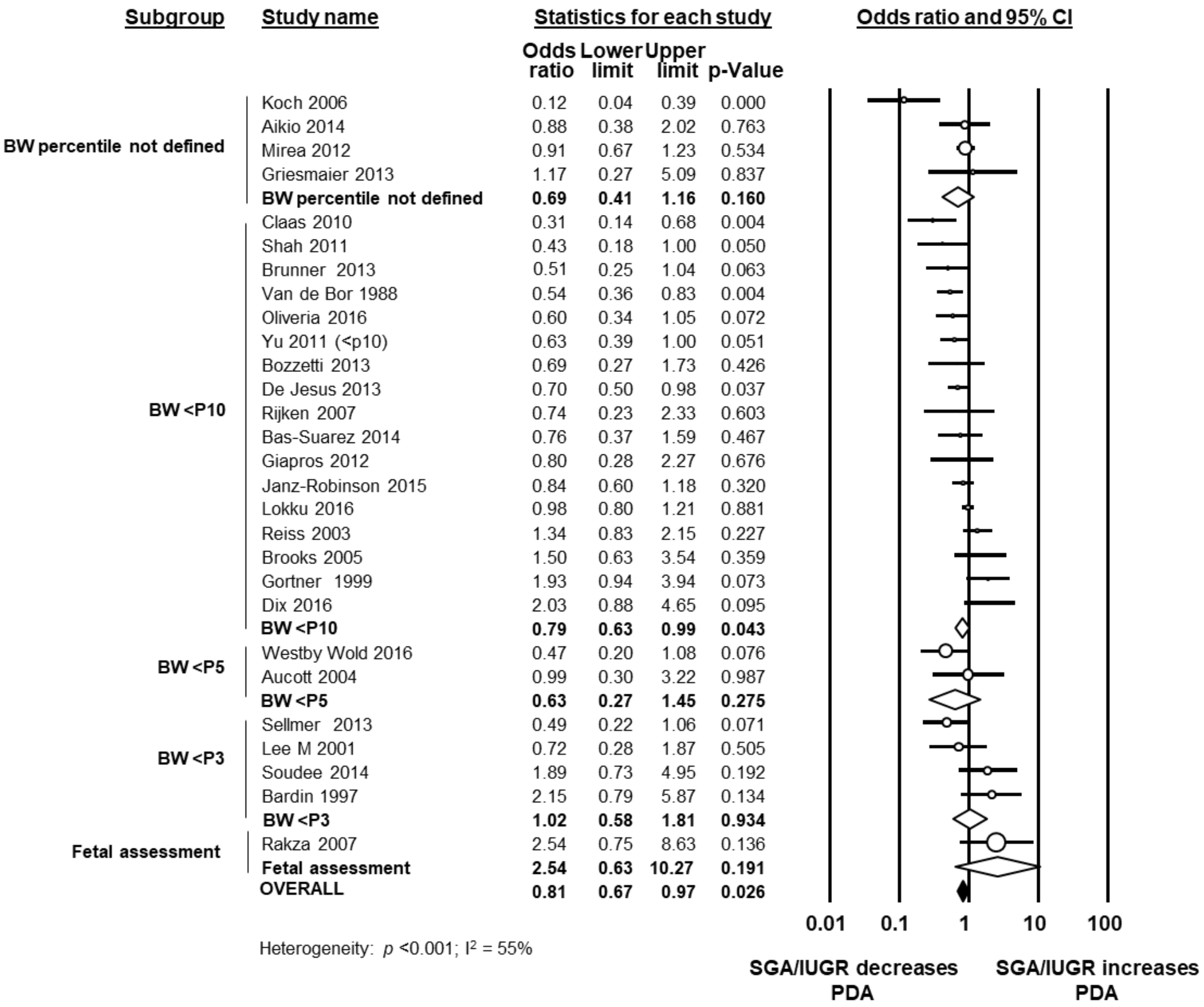

Supplementary Figure 5. Subgroup meta-analysis on the association between growth restriction and patent ductus arteriosus (PDA) of studies which only included infants with gestational age <28 weeks or birth weight (BW) <1000g. CI: confidence interval; SGA: small for gestational age; IUGR: intrauterine growth restriction; <P10: BW lower than 10th percentile; <P5: BW lower than 5th percentile; <P3: BW lower than 3rd percentile.

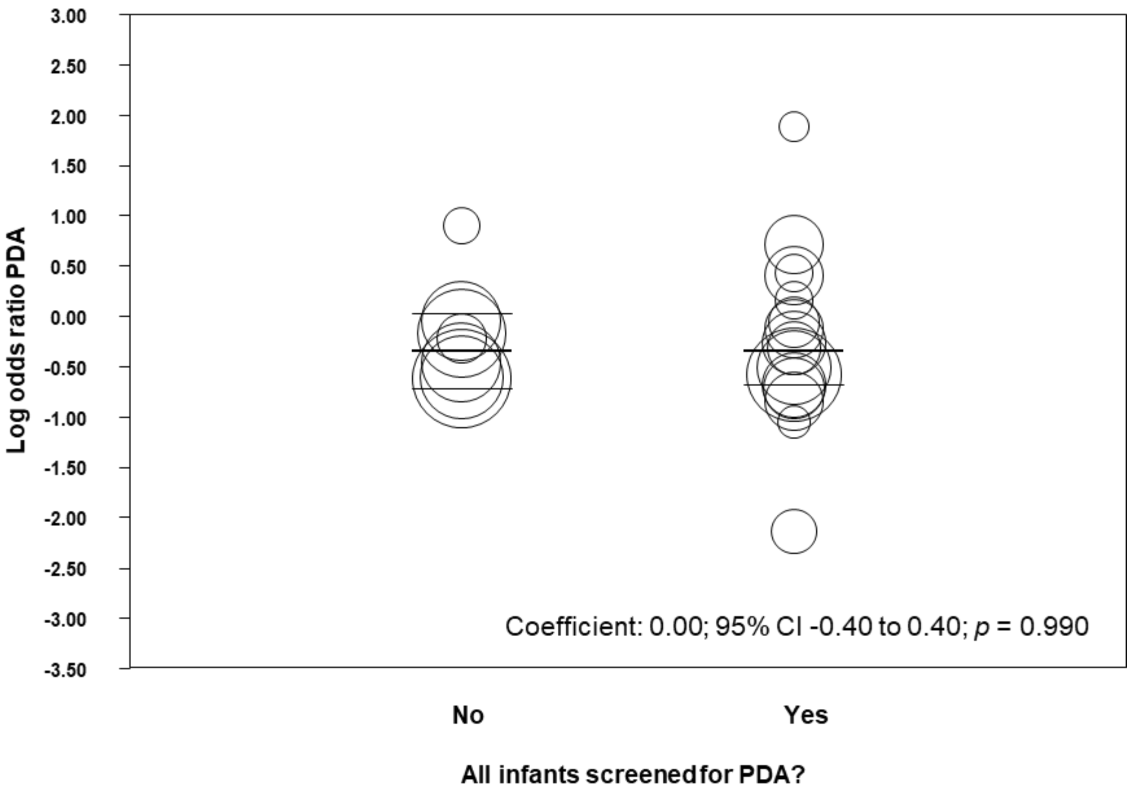

Supplementary Figure 6. Meta-regression comparing studies which screened all infants for PDA vs. studies which only assessed PDA in selected infants. PDA: patent ductus arteriosus; CI: confidence interval.

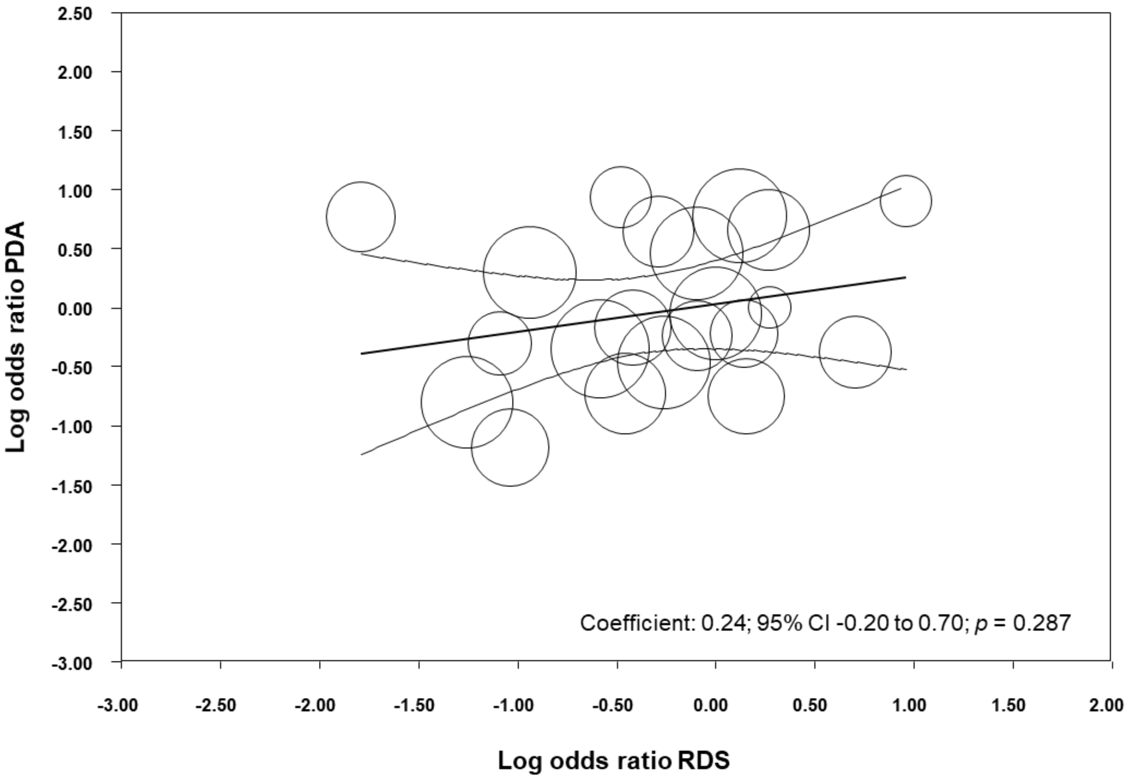

Supplementary Figure 7. Meta-regression of risk of patent ductus arteriosus (PDA) in the growth restricted group and risk of respiratory distress syndrome (RDS). CI: confidence interval.
